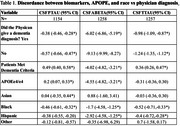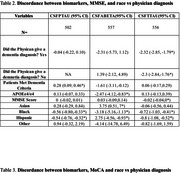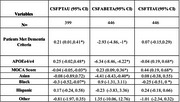# Dementia Diagnostic Confidence Among Ethnoculturally Diverse Minorities

**DOI:** 10.1002/alz70856_098804

**Published:** 2025-12-24

**Authors:** Chidera Agwu, Charles C. Windon, Thomas J. Hoffmann

**Affiliations:** ^1^ Washington University School of Medicine in St. Louis, St. Louis, CO, USA; ^2^ Department of Neurology, University of California, San Francisco, San Francisco, CA, USA; ^3^ University of California San Francisco, Department of Epidemiology and Biostatistics, San Francisco, CA, USA

## Abstract

**Background:**

As the aging population increases, Alzheimer's prevalence increases, and it disproportionally affects people from diverse backgrounds. Proper diagnosis and detection are essential for treating patients with cognitive deficits and dementia due to Alzheimer's disease. There is evidence that supports some of the leading causes of these findings are systemic barriers such as the location of health providers and healthcare interactions. To answer this question, we want to examine the disagreement between objective measures of cognitive impairment and Alzheimer's biomarkers. We also want to examine how providers affect the relationship between diagnosis and biomarkers.

**Method:**

Using data from the NACC (National Alzheimer's Coordinating Center) we received data for patient information that included factors such as race (Black, Asian, Latino, White, and Hispanic), MMSE and MOCA scores, biomarkers (CSF Amyloid‐Beta, CSF Total Tau, and CSF Phospho‐Tau), etc. We will use a regression analysis to look at the association between MOCA or MMSE and biomarkers across the different racial groups. Next, we will use a regression analysis to look at the association between clinical diagnosis and biomarkers across the different racial groups compared to White patients.

**Result:**

We have the total number of patients (*n* = 180,039) of those patients 24.5% identify as minorities that have biomarker data (*n* = 3050). Black (‐0.46, ‐1.7, ‐0.52; *p* <0.05) and Hispanic (‐0.38, ‐2.92, ‐0.4; *p* <0.05) patients show high levels of discordance with physician diagnosis across all biomarkers (Table 1). Looking at MMSE and physician diagnosis there are similar significant results with Black (‐0.56, ‐3.18, ‐0.72; *p* <0.05) and Hispanic (‐0.56, 2.75, ‐0.8; *p* <0.05) patients as well (Table 2). MoCA is significant across all three biomarkers showing that it influences minorities who are considered demented ( ‐0.04, 0.23, 0.44; *p* > 0.05) (Table 3).

**Conclusion:**

We see that certain minority groups have a higher chance of not being properly diagnosed with dementia, and the chance of that increases when certain cognitive tests are used. The testing measures presented here are influenced by other factors, studying the factors could potentially lead to another study. The implications of this study show that testing measures and physician's diagnoses need to account for diverse individuals.